# An explainable machine learning framework for accurate prediction of postoperative anterior chamber depth in highly myopic cataract surgery

**DOI:** 10.3389/fcell.2026.1876904

**Published:** 2026-07-07

**Authors:** Yuyang Yang, Haoqiang Cui, Jiajia Gao, Yuqing Wang, Beiou Zhang, Jiahao Liu, Honghua Yu, Wenjie Wu, Li Li

**Affiliations:** 1 Department of Ophthalmology, Shengli Clinical Medical College of Fujian Medical University, Fujian Provincial Hospital, Fuzhou University Affiliated Provincial Hospital, Fuzhou, China; 2 Department of Ophthalmology and Optometry, Fujian Medical University, Fuzhou, China; 3 School of Basic Medical Sciences, Fujian Medical University, Fuzhou, China; 4 Institute for Health and Sport, Victoria University, Melbourne, VIC, Australia; 5 Centre for Eye Research Australia, Royal Victorian Eye and Ear Hospital, East Melbourne, VIC, Australia; 6 Ophthalmology, Department of Surgery, The University of Melbourne, Melbourne, VIC, Australia; 7 Joint Shantou International Eye Center of Shantou University and the Chinese University of Hong Kong, Shantou, Guangdong, China; Guangdong Provincial People’s Hospital (Guangdong Academy of Medical Sciences), Southern Medical University, Guangzhou, China

**Keywords:** anterior chamber depth, cataract, high myopia, machine learning, prediction model

## Abstract

**Purpose:**

To develop and validate machine learning models for predicting postoperative anterior chamber depth (ACD) in highly myopic cataract patients based on preoperative biometric parameters.

**Methods:**

This prospective study enrolled 203 eyes of 127 highly myopic patients who underwent phacoemulsification and intraocular lens (IOL) implantation between January 2024 and December 2025. Ocular biometric parameters were measured preoperatively and at 3 months postoperatively. A dual feature selection strategy combining Least Absolute Shrinkage and Selection Operator (LASSO) regression and Boruta algorithm was employed to identify important predictors of postoperative ACD in these samples. We compared five machine learning algorithms and evaluated their performance using the coefficient of determination (*R* ^2^), mean absolute error (MAE), root mean square error (RMSE), and accuracy within ±0.1 mm and ±0.2 mm. Subsequently, Shapley Additive Explanations (SHAP) method was applied to interpret the optimal model’s feature importance.

**Results:**

Six predictors were identified for model construction: axial length ACD/lens thickness ratio (ACD/LT), white-to-white distance (WTW), ACD at 90° (ACD90), horizontal position angle, and ACD + LT/2. Among all models, Random Forest algorithm demonstrated the best predictive performance, achieving an *R* ^2^ of 0.8259, mean absolute error of 0.0604 mm, and root mean square error of 0.0722 mm in the test set. The accuracy within ±0.1 mm reached 80.49%, and within ±0.2 mm reached 100%. SHAP analysis revealed that ACD + LT/2 was the most important predictor, followed by WTW, ACD90, and horizontal position angle.

**Conclusion:**

This study successfully developed and validated an explainable RF-based machine learning model for the prediction of postoperative ACD in highly myopic cataract patients, which supports more reliable IOL power calculation and offers a practical tool for optimizing surgical planning in highly myopic eyes.

## 1 Introduction

High myopia was defined as a spherical equivalent (SE) ≤ −6.00 diopters (D), often accompanied by an axial length (AL) exceeding 26.0 mm, is projected to affect 9.8% of the global population by 2050 (Holden et al., 2016). High myopia is significantly associated with prevalent nuclear and posterior subcapsular cataract (Pan et al., 2013). Advances in ocular biometry and intraocular lens (IOL) formulas have transformed cataract surgery from a purely vision-restoring procedure into a refractive one—a shift that holds particular significance for patients with high myopia. In this population, cataract surgery serves a dual purpose: addressing lens opacity while simultaneously correcting pre-existing refractive error. Moreover, given the well-established association between high myopia and higher educational attainment (Shan et al., 2022), these patients often have heightened expectations for postoperative visual outcomes. Consequently, achieving precise postoperative refraction prediction has become imperative, as even minor errors in IOL power calculation can substantially compromise visual quality and patient satisfaction.

Anterior chamber depth (ACD) is a critical parameter determining the effective lens position (ELP), defined as the distance from the anterior corneal surface to the principal plane of IOL, and its postoperative changes directly influence refractive prediction accuracy (Li et al., 2020). In the general population, various regression formulas have been developed for postoperative ACD prediction (Li et al., 2020; Yoo et al., 2019; Lou et al., 2024; Mita et al., 2024; Hoffer and Savini, 2020). However, highly myopic eyes exhibit distinctive anatomical characteristics, including significant axial elongation, increased lens thickness, and altered scleral biomechanical properties (Jonas et al., 2022). These features may result in different patterns of postoperative ACD changes compared to non-myopic eyes, necessitating population-specific prediction models.

In recent years, artificial intelligence technologies have been increasingly adopted in ophthalmology and have shown broad applicability in disease screening, diagnostic support, treatment response prediction, and medical education (Li et al., 2025; Yuan et al., 2025; Cao et al., 2025). Among these technologies, machine learning approaches have demonstrated considerable promise in handling complex nonlinear relationships and extracting informative features from high-dimensional data, thereby improving the accuracy of clinical prediction models (Lu et al., 2025; Ting et al., 2021). However, machine learning studies specifically focused on postoperative ACD prediction in highly myopic patients remain limited. In this study, we developed and validated machine learning models for predicting postoperative ACD in highly myopic cataract patients using preoperative biometric parameters. We employed a dual feature selection strategy to identify core predictors, compared five machine learning algorithms, and applied SHAP analysis to enhance model interpretability.

## 2 Methods

### 2.1 Study population

This prospective case series consecutively enrolled highly myopic patients who underwent cataract surgery at the Department of Ophthalmology, Fujian Provincial Hospital, from January 2024 to December 2025. Inclusion criteria were: (1) age ≥18 years; (2) AL ≥ 26 mm; (3) uneventful phacoemulsification with in-the-bag implantation of TECNIS ZCB00 IOLs (Johnson and Johnson Vision, Santa Ana, CA, USA) or Proming A1-UV IOLs (Eyebright Medical Technology, Beijing, China); and (4) availability of complete preoperative biometric parameters and postoperative IOL-Master 700 measurements at 3 months. Exclusion criteria included a history of other ocular surgery, previous trauma, strabismus, zonular weakness, severe fundus pathology, severe intraoperative or postoperative complications, or poor quality of anterior segment imaging that precluded accurate assessment.

Ethics approval was obtained from the Ethics Committee of Fuzhou University Affiliated Provincial Hospital (ID: K2023–12–012). Written informed consent was obtained from all participants. This study followed the tenets of the Declaration of Helsinki.

### 2.2 Data collection and measurements

All patients underwent a comprehensive ophthalmological examination before surgery. Preoperative evaluation included the assessment of visual acuity, intraocular pressure, slit-lamp examination, funduscopy, and B-scan ultrasonography. The spherical power of the IOL was calculated with the Barrett Universal II formula.

Biometric parameters, including AL, ACD (measured from the corneal epithelium to the lens), corneal curvature (K1, K2), central corneal thickness (CCT), lens thickness (LT), and white-to-white distance (WTW), were measured using the IOLMaster 700 (Carl Zeiss Meditec AG, Jena, Germany) preoperatively and at 3 months postoperatively.

Corneal topographic parameters, including higher-order aberrations (HOAs), corneal asphericity coefficient (Q-value), corneal eccentricity (e), surface regularity index (SRI), and surface asymmetry index (SAI), were obtained using the OPD-Scan III (Nidek Co., Ltd., Gamagori, Japan) preoperatively. Spherical equivalent (SE), photopic pupil diameter (PPD), and mesopic pupil diameter (MPD) were also measured using the same device.

ACD measurements at four orientations (0°, 90°, 180°, 270°), central ACD, chamber volume (CV), and horizontal position angle (defined as the horizontal offset angle of the pupil center relative to the corneal vertex) were obtained using the Pentacam AXL (Oculus Optikgeräte GmbH, Wetzlar, Germany) preoperatively.

Based on the raw measurements, the following derived parameters were calculated: the ratio of ACD to AL (ACD/AL), the ratio of central corneal thickness to AL (CCT/AL), the ratio of ACD to lens thickness (ACD/LT), the ratio of AL to mean corneal curvature (AL/K), and the sum of ACD and half of LT (ACD + LT/2), which represents a rough estimate of the lens position.

### 2.3 Feature selection

To enhance model robustness and reduce multicollinearity, a dual feature selection strategy combining linear and non-linear approaches was employed. First, Least Absolute Shrinkage and Selection Operator (LASSO) regression with L1 regularization was applied, using 10-fold cross-validation to select the optimal regularization parameter (λ). Variables with non-zero coefficients were retained for preliminary dimensionality reduction. Second, the Boruta algorithm based on Random Forest (RF) was employed for comprehensive feature selection. Through 500 iterations, the Z-scores of original features were compared with those of random shadow features to identify features with significant predictive value. Only features identified by both LASSO and Boruta algorithms were included in the final model construction. This strategy aimed to extract core anatomical parameters highly associated with postoperative ACD through mutual validation, minimizing the risk of model overfitting.

### 2.4 Model development and evaluation

Based on the R language caret package and related machine learning frameworks, five regression models were developed and compared: Elastic Net, Support Vector Machine (SVM), RF, XGBoost, and LightGBM. The dataset was randomly split into a training set (80%, n = 162) and a test set (20%, n = 41) according to the original sample order for model development and generalization performance evaluation, respectively. To further evaluate the potential influence of inter-eye correlation, a sensitivity analysis was performed using only one randomly selected eye from each patient. The Random Forest model was retrained using the same predictors and evaluated with the same performance metrics.

To optimize model performance, grid search strategy was employed for hyperparameter tuning. During training, 3-repeat 10-fold cross-validation was used to evaluate the stability of parameter configurations. For ensemble learning models such as XGBoost and LightGBM, randomized grid search was further applied to optimize core parameters including tree depth, learning rate, sample and feature sampling rates. The optimal parameter combination was selected based on minimizing the root mean square error (RMSE) in the validation set. For the Random Forest model, hyperparameter tuning was performed using grid search. The number of trees was fixed at 1,000, while the mtry parameter was optimized over a range of 2–6. The optimal parameter combination was selected according to the lowest RMSE obtained during cross-validation.

Model performance was evaluated using coefficient of determination (*R* ^2^), mean absolute error (MAE), and RMSE. Additionally, clinical accuracy metrics were calculated: accuracy within ±0.1 mm (Acc_0.1 mm) and ±0.2 mm (Acc_0.2 mm) error ranges. Bland-Altman plots were generated to assess agreement between actual and predicted values.

Shapley Additive Explanations (SHAP) was calculated to quantify global and individual feature contributions, enhancing the model transparency and clinical interpretability of the machine learning. SHAP summary plots and waterfall plots were generated to identify core predictors and elucidate individualized decision pathways.

### 2.5 Statistical analysis

Continuous variables were first assessed for normality using the Shapiro–Wilk test. Normally distributed variables were expressed as mean ± standard deviation (SD), and non-normally distributed variables as median with interquartile range (IQR). Intergroup comparisons were performed using the independent samples t-test or Mann-Whitney U test as appropriate. Categorical variables were compared using the chi-square test or Fisher’s exact test. All of the statistical analyses were performed in R 4.5.0, and a two-sided p value <0.05 was considered to indicate statistical significance.

## 3 Result

### 3.1 Baseline characteristics

A total of 203 eyes from 127 patients were enrolled, with 76 patients contributing both eyes (152 eyes) and 51 patients contributing one eye (51 eyes). The dataset was randomly split at the patient level into a training set (102 patients, 162 eyes) and a test set (25 patients, 41 eyes) at an approximate 8:2 ratio, ensuring that both eyes from the same patient were assigned to the same set. The baseline characteristics of both sets are summarized in Table 1. Most key parameters were well-balanced between the two groups, although significant differences were noted in age, HOAs, and SAI (all P < 0.05). Regarding surgical outcomes for the entire cohort, the postoperative ACD increased by a mean of 1.58 mm compared to preoperative measurements.

**TABLE 1:** Baseline characteristics of the study population in the training and testing sets.

Variable	Training set N = 162	Test set N = 41	P_value
Age (years)	63 [55, 71.5]	67 [62, 72]	<0.05
Gender (male = 0, female = 1)	0 [0, 1]	0 [0, 1]	0.656
AL (mm)	28.015 [26.678, 29.805]	27.34 [26.64, 29.21]	0.45
ACD/AL	0.119 ± 0.016	0.121 ± 0.015	0.681
CCT (mm)	538.259 ± 36.326	546.951 ± 32.515	0.14
CCT/AL	18.991 ± 1.904	19.572 ± 1.667	0.057
ACD (mm)	3.4 [3.12, 3.607]	3.4 [3.01, 3.68]	0.837
LT (mm)	4.33 ± 0.438	4.428 ± 0.365	0.146
ACD/LT	0.787 [0.684, 0.889]	0.782 [0.654, 0.848]	0.423
K1 (dioptre)	43.675 [42.605, 44.72]	42.98 [42.56, 43.88]	0.125
K2 (dioptre)	44.57 [43.335, 45.828]	43.8 [43.09, 45.03]	0.133
K (dioptre)	44.063 ± 1.661	43.744 ± 1.769	0.301
AL/K	0.643 [0.604, 0.688]	0.628 [0.609, 0.671]	0.734
WTW (mm)	11.9 [11.6, 12.2]	11.9 [11.5, 12.2]	0.996
SE (dioptre)	−8.25 [-10.812, −6.25]	−7.25 [-9.875, −6.125]	0.569
HOA (um)	0.158 [0.121, 0.208]	0.169 [0.144, 0.249]	<0.05
Q	−0.125 [-0.21, −0.02]	−0.06 [-0.26, 0.01]	0.336
e	0.35 [0.135, 0.467]	0.25 [-0.11, 0.51]	0.342
SRI	0.435 [0.29, 0.69]	0.5 [0.4, 0.74]	0.107
SAI	0.38 [0.33, 0.45]	0.4 [0.36, 0.53]	<0.05
PPD (mm)	3.35 [3.02, 3.755]	3.4 [3.08, 3.73]	0.823
MPD (mm)	5.041 ± 0.912	4.784 ± 0.789	0.076
ACD90 (mm)	1.89 [1.55, 2.07]	1.82 [1.49, 2.07]	0.686
ACD0 (mm)	2.09 [1.81, 2.357]	1.99 [1.71, 2.33]	0.173
ACD270 (mm)	2.16 [1.89, 2.42]	2.11 [1.77, 2.47]	0.431
ACD180 (mm)	2.06 [1.8, 2.332]	2.03 [1.74, 2.54]	0.732
Central ACD (mm)	2.925 [2.69, 3.198]	2.94 [2.65, 3.38]	0.658
CV (mm3)	159 [127.5, 181.75]	155 [118, 184]	0.366
Horizontal position angle (degree)	34.65 [31.325, 39.075]	34.4 [28.5, 38.1]	0.288
ACD + LT/2	5.551 ± 0.311	5.585 ± 0.308	0.532
Postop_ACD (mm)	4.94 [4.76, 5.16]	4.98 [4.86, 5.09]	0.513

Abbreviations: AL, axial length; ACD, anterior chamber depth; CCT, central corneal thickness; LT, lens thickness; K = corneal curvature; WTW, white-to-white distance; SE, spherical equivalent; HOA, higher-order aberrations; Q = corneal asphericity coefficient; e = corneal eccentricity; SRI, surface regularity index; SAI, surface asymmetry index; PPD, photopic pupil diameter; MPD, mesopic pupil diameter; CV, chamber volume.

### 3.2 Equations feature selection

**FIGURE 1:**
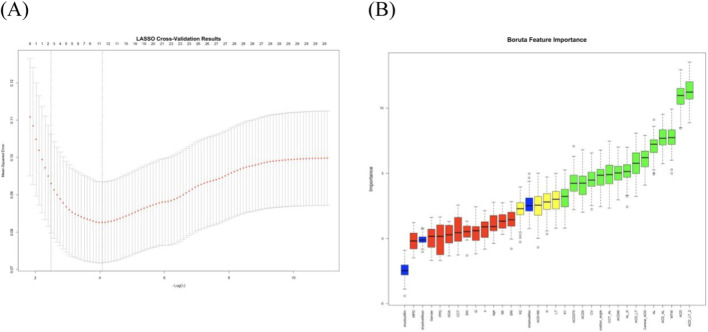
Feature selection process using LASSO regression and the Boruta algorithm. **(A)** LASSO coefficient selection via 10-fold cross-validation. **(B)** Feature importance ranking based on the Boruta algorithm. Abbreviations: AL = axial length; ACD = anterior chamber depth; CCT = central corneal thickness; LT = lens thickness; K = corneal curvature; WTW = white-to-white distance; SE = spherical equivalent; HOA = higher-order aberrations; Q = corneal asphericity coefficient; e = corneal eccentricity; SRI = surface regularity index; SAI = surface asymmetry index; PPD = photopic pupil diameter; MPD = mesopic pupil diameter; CV = chamber volume.

To identify the most relevant predictors of postoperative ACD and reduce model redundancy, two machine learning algorithms were employed for feature selection. First, LASSO regression with 10-fold cross-validation identified 11 variables with non-zero coefficients (Figure 1A): AL, CCT, ACD/LT, K2, WTW, e, SAI, ACD90, ACD180, horizontal position angle, and ACD + LT/2. Second, the Boruta algorithm was utilized to identify 15 important features through 500 iterations (Figure 1B), including AL, ACD/AL, CCT/AL, ACD, ACD/LT, K1, AL/K, WTW, ACD90, ACD0, ACD270, Central/ACD, CV, horizontal position angle, and ACD + LT/2. By taking the intersection of these two sets, six core predictors were ultimately determined as the input features for subsequent model construction: AL, ACD/LT, WTW, ACD90, horizontal position angle, and ACD + LT/2.

### 3.3 Model comparison and evaluation

**FIGURE 2:**
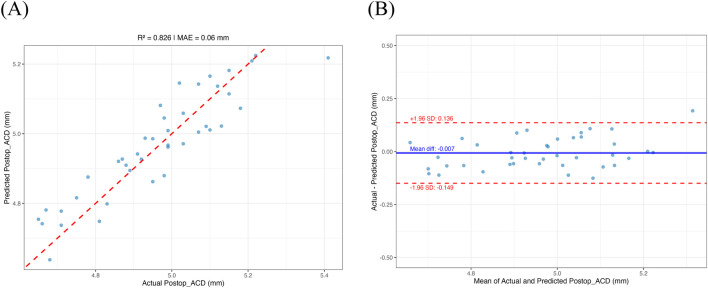
Evaluation of predictive performance and agreement between actual and predicted postoperative ACD. **(A)** Prediction accuracy and correlation analysis. **(B)** Bland-Altman plot for agreement between actual and predicted Postop_ACD. Abbreviations: ACD = anterior chamber depth; *R* ^2^ = coefficient of determination; MAE = mean absolute error.

The predictive performance of the five machine learning algorithms on the test set is compared in Table 2. RF demonstrated the best performance, achieving an *R* ^2^ of 0.8259, with the lowest MAE (0.0604 mm) and RMSE (0.0722 mm) (Figure 2A). Regarding prediction accuracy, RF achieved 80.49% accuracy within ±0.1 mm, and all models exhibited high accuracy (97.56%–100%) within ±0.2 mm. Bland-Altman analysis showed good agreement between actual and predicted values for RF, with 95% limits of agreement from −0.15 to +0.14 mm (Figure 2B). In comparison, SVM, ElasticNet, and XGBoost demonstrated comparable predictive performance, with *R* ^2^ values of 0.6983, 0.6941, and 0.6847, and corresponding MAE values of 0.0781, 0.0776, and 0.0787, respectively. In contrast, LightGBM exhibited a relatively higher prediction error (MAE = 0.0860). Taking all evaluation metrics into account, the RF model outperformed the other algorithms and was identified as the optimal predictive model for this study. To further assess the potential influence of inter-eye correlation, a sensitivity analysis was conducted using only one randomly selected eye from each patient. The Random Forest model achieved an *R* ^2^ of 0.7677, MAE of 0.1051 mm, RMSE of 0.1364 mm, with accuracies of 60.00% within ±0.1 mm and 88.00% within ±0.2 mm (Supplementary Figure 1).

**TABLE 2:** Performance comparison of different machine learning models in the testing set.

Model	R2	MAE	RMSE	Acc_0.1 mm	Acc_0.2 mm
Random forest	0.8259	0.0604	0.0722	0.8049	1.0000
SVM	0.6983	0.0781	0.0951	0.6829	1.0000
ElasticNet	0.6941	0.0776	0.0958	0.7073	0.9756
XGBoost	0.6847	0.0787	0.0972	0.7073	1.0000
LightGBM	0.6627	0.0860	0.1005	0.6341	0.9756

Abbreviations: SVM, support vector machine; *R* ^2^ = coefficient of determination; MAE, mean absolute error; RMSE, root mean square error.

### 3.4 Model interpretability and clinical application

**FIGURE 3:**
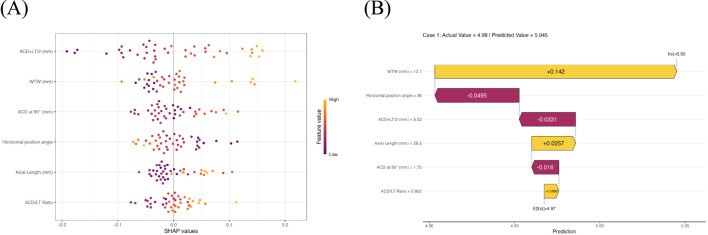
Visual interpretation of the Random Forest model using SHAP analysis. **(A)** Global explanation of feature contribution based on SHAP beeswarm plot. **(B)** Local interpretation of a specific case using SHAP waterfall plot. Abbreviations: SHAP = Shapley Additive Explanations; ACD = anterior chamber depth; LT = lens thickness; WTW = white-to-white distance.

SHAP analysis provided in-depth interpretation of the optimal RF model (Figure 3). The SHAP summary plot (Figure 3A) revealed that ACD + LT/2 was the most critical predictor of postoperative ACD, exhibiting the widest distribution of SHAP values. Following important features included WTW, ACD90, horizontal position angle, AL, and ACD/LT ratio. Regarding contribution direction, specifically, ACD + LT/2, WTW, ACD90, AL, and the ACD/LT contributed positively to the predicted postoperative ACD, whereas horizontal position angle exhibited a negative contribution.

In this study, a SHAP waterfall plot was employed to perform an individualized feature attribution analysis for a randomly selected subject (Figure 3B). In this case, the observed postoperative value was 4.98 mm, while the model predicted 5.045 mm, demonstrating excellent agreement and high predictive accuracy. Quantitative decomposition of feature contributions showed that the relatively large white-to-white distance (WTW = 12.7 mm) exerted the strongest positive effect on the prediction (contribution: +0.142). In contrast, the horizontal position angle (36°), ACD + LT/2 (5.52 mm), and ACD at 90° (1.75 mm) served as the primary negative contributors, reducing the predicted value by −0.0495, −0.0331, and −0.016, respectively. Additionally, axial length (28.5 mm) and the ACD/LT ratio (0.802) provided modest positive contributions. This example quantitatively illustrates how multiple anatomical parameters, through synergistic and antagonistic interactions, jointly determine the predicted postoperative anterior chamber depth in patients with high myopia.

## 4 Discussion

Cataract remains one of the leading causes of visual impairment worldwide. With increasing patient expectations for postoperative visual quality and refractive outcomes, improving the accuracy of IOL power calculation has become a major focus in cataract surgery. In recent years, AI has rapidly advanced in ophthalmology and has shown considerable potential in disease diagnosis, risk prediction, and clinical outcome assessment (Gong et al., 2023; Yi et al., 2024; Zhao et al., 2022). In the field of cataract surgery, AI-based approaches have also provided new opportunities to improve the accuracy of IOL power calculation and optimize postoperative refractive outcomes (Zhu et al., 2023). Accurate IOL power calculation is essential for optimal refractive outcomes in cataract surgery. Utilizing thick-lens ray-tracing and a partial derivative mathematical model, Sverker Norrby identified that postoperative IOL position prediction (35%), postoperative refraction (27%), and AL measurement (17%) are the three primary sources of refractive error, collectively accounting for 79% of the total error (Norrby, 2008). As a critical input parameter in IOL formulas, the accuracy of the ELP prediction is a primary determinant of postoperative refractive success (Retzlaff et al., 1990; Sheard et al., 2010; Haigis et al., 2000). Particularly in highly myopic eyes, the postoperative ACD closely corresponds to the ELP; thus, enhancing the predictive precision of postoperative ACD is key to refining IOL calculation accuracy in this population. In our study, we have presented a machine learning approach for predicting postoperative ACD in highly myopic cataract patients using standard preoperative optical biometry measurements. Through a dual feature selection strategy combining LASSO and Boruta algorithms, we identified six core predictors: AL, ACD/LT, WTW, ACD90, horizontal position angle, and ACD + LT/2. Among five machine learning algorithms evaluated, RF demonstrated superior predictive performance, achieving an *R* ^2^ of 0.8259 and MAE of 0.0604 mm in the test set, 80.49% of the postoperative ACD prediction errors fell within ±0.1 mm. Although slight baseline differences in age, HOA, and SAI were observed between the training and test sets following random data splitting, these variables were not retained in the final model, suggesting that such differences are unlikely to substantially affect model generalizability.

SHAP analysis identified ACD + LT/2 as the most significant predictor. This parameter represents the distance from the anterior corneal surface to the center of the crystalline lens, roughly reflecting the axial position of the lens within the eye. Its positive contribution indicates that a larger preoperative ACD + LT/2 value predicts a deeper postoperative ACD. This relationship is consistent with previous findings suggesting that preoperative ACD and LT are determinative factors for the ELP of an IOL. The study by Ning et al. demonstrated that the postoperative ACD exhibits a positive correlation with both the preoperative ACD and the composite parameter preoperative ACD + LT/2 (Ning et al., 2019). Furthermore, ACD/LT contributes positively to the prediction of postoperative ACD. This ratio reflects the proportional distribution of the ACD relative to the LT within the anterior segment, thereby indirectly indicating the preoperative lens position. A higher ACD/LT ratio indicates a greater proportion of ACD relative to the lens, which is positively correlated with a deeper postoperative ACD (Ning et al., 2019; Jeong et al., 2017; Olsen, 2006). WTW ranked second in importance. As a surrogate for ciliary sulcus diameter, WTW directly influences IOL haptic positioning (Vanathi, 2024; Werner et al., 2004). Multiple large-scale cohorts have demonstrated that a larger WTW diameter is associated with a deeper anterior chamber, exhibiting a moderate positive correlation. This positive correlation between ACD and WTW persists in both normal and cataractous populations, while being concurrently influenced by AL, lens thickness, and age. These findings indirectly demonstrate that WTW makes a positive contribution to the prediction of postoperative ACD (Xu et al., 2021; Lei et al., 2021; Wei et al., 2021). Additionally, the study by Shen also demonstrated that WTW and AL are positively correlated with ELP (Shen et al., 2024). ACD90 and the horizontal position angle are novel predictors that reflect zonular tension distribution and optical-anatomical centration, respectively. Highly myopic eyes often exhibit zonular laxity and posterior staphyloma, leading to asymmetrical capsular bag support (Jonas et al., 2022). The positive contribution of ACD90 and the negative contribution of horizontal position angle suggest that these parameters capture individual variations in ocular geometry that influence IOL position. To our knowledge, this is the first study to incorporate these Pentacam-derived parameters into a machine learning model for ACD prediction.

The dual feature selection strategy (LASSO + Boruta) is a strength of this study. LASSO effectively handles multicollinearity by shrinking coefficients of correlated predictors (Tibshirani, 2011; Saputro et al., 2025), while Boruta identifies features with true predictive value by comparing with random shadow features (Ejiyi et al., 2024). This combined approach ensures that only robust, non-redundant predictors enter the final model, reducing overfitting risk—a critical consideration given our sample size (n = 203). The use of SHAP analysis significantly enhances model interpretability. Unlike traditional black-box machine learning models, SHAP values provide both global (which features matter most) and local (why a specific prediction was made) explanations (Ponce-Bobadilla et al., 2024). Beyond identifying important predictors, the explainability of the proposed model has important clinical implications. By incorporating the SHAP framework, our model not only provides accurate predictions of postoperative ACD but also quantifies the contribution of each biometric parameter to the prediction outcome. At the global level, SHAP analysis demonstrated that ACD + LT/2, WTW, and ACD90 were among the most influential predictors of postoperative ACD. These predictors are largely consistent with the current understanding of ELP and anterior segment anatomy, suggesting that the model’s decision-making process is supported by biologically plausible mechanisms. At the local level, SHAP waterfall plots illustrate the positive or negative contribution of individual features to the predicted postoperative ACD for each patient, enabling clinicians to understand the rationale underlying a specific prediction rather than relying solely on a predicted value. Such a transparent prediction process may enhance clinicians’ trust in artificial intelligence models and improve their acceptability in real-world clinical practice (Ting et al., 2018).

Recent years have witnessed the emergence of AI-driven IOL formulas such as Kane, RBF 3.0, Zhu-Lu, and Hoffer QST, which have shown improved accuracy in highly myopic eyes (Cheng et al., 2020; Guo et al., 2023; Taroni et al., 2023). However, these formulas predict refractive outcome (spherical equivalent) rather than the anatomical ACD. Our model directly predicts postoperative ACD, which can be used as an input for ray-tracing or thick-lens IOL calculations. Moreover, our model is transparent and does not rely on proprietary algorithms, making it adaptable to different IOL designs and clinical settings. The study by Shen showed that in patients with AL ≥ 26 mm, a 1 mm error in ELP would result in a refractive error of 0.54 D (Shen et al., 2024). Our RF model demonstrates high accuracy, with a MAE of 0.0604 mm, which is expected to significantly improve postoperative refractive outcomes in highly myopic cataract patients. It also shows a certain advantage compared with the accuracy reported by leading AI-driven formulas: Kane (MAE of approximately 0.33 D for highly myopic eyes) (Vilaltella et al., 2025), RBF 3.0 (MAE of 0.30 D in long eyes) (Mo et al., 2024), Hoffer QST (MedAE of 0.19 D, RMSAE of 0.351 D) (Mo et al., 2023), and Zhu-Lu (MedAE of approximately 0.30 D; RMSAE of 0.436 D for plate-haptic IOLs) (Guo et al., 2023; Mo et al., 2024).

Accurate prediction of postoperative ACD has direct clinical utility. First, it improves IOL power calculation by refining ELP estimates, particularly in eyes with extreme ALs or atypical anterior segment anatomy. Second, the model can help identify eyes that are predicted to have unusually shallow or deep postoperative ACD, alerting surgeons to consider alternative IOL types or surgical techniques (e.g., capsular tension rings (Qi et al., 2023)) in selected cases. Third, the six identified predictors are routinely measured with standard optical biometry and corneal topography (IOLMaster, Pentacam, OPD), requiring no additional equipment or expertise. This facilitates seamless integration into existing preoperative workflows. More importantly, our model goes beyond simple linear regression by incorporating non-linear interactions and additional anatomical features (ACD/LT ratio, WTW, ACD90, horizontal position angle), offering a substantial improvement in predictive accuracy that can directly translate into fewer refractive surprises. Notably, keratometry was not selected as a core predictor in our final model, suggesting that precise postoperative ACD prediction can be achieved without relying on corneal curvature measurements—a feature that may enhance the model’s applicability in post-refractive surgery populations. Although our study did not include such patients, the absence of keratometry in the predictor set indicates that the model could theoretically perform well even when corneal power data are unavailable or inaccurate. Future prospective validation in patients with prior refractive surgery is warranted to confirm this hypothesis.

This study has several limitations. First, this study included bilateral eyes from some patients. Although a patient-level data-splitting strategy was adopted to prevent information leakage between the training and test sets, inter-eye correlation may still have had some influence on model performance. A sensitivity analysis using only one eye per patient showed a modest reduction in predictive performance compared with the primary analysis, which may be attributable to the reduced sample size. Nevertheless, the model maintained good predictive ability, supporting the robustness of the study findings. Second as a single-center study with a relatively modest sample size, it may be subject to selection bias, and the generalizability of the findings requires validation in larger, multicenter cohorts. Third, biometric data were obtained from three different devices; although each provides high-quality measurements, potential inter-device variability cannot be excluded. Future studies using standardized measurements from a single platform may help minimize this source of variability. Finally, direct comparison with existing IOL formulas for anterior chamber depth prediction was not performed, as these formulas are typically integrated within proprietary calculation software and do not provide standalone ACD outputs.

## 5 Conclusion

We developed and validated an explainable random forest model for predicting postoperative anterior chamber depth in highly myopic eyes. Based solely on routinely available preoperative biometric parameters, the model provides transparent, patient-specific interpretability through SHAP analysis. Its integration into the preoperative workflow may improve intraocular lens power selection, reduce refractive uncertainty, and support more precise and personalized cataract surgery in the growing population of patients with high myopia.

## Data Availability

The raw data supporting the conclusions of this article will be made available by the authors, without undue reservation.

## References

[B1] Cao D. Yao J. Ting D. S. W. Tan G. S. W. Cao D. Yao J. (2025). Artificial intelligence in predicting anti-VEGF treatment response in diabetic macular edema: current progress and future directions. VNS 42 (1), 0. 10.48130/vns-0025-0027

[B2] Cheng H. Wang L. Kane J. X. Li J. Liu L. Wu M. (2020). Accuracy of artificial intelligence formulas and axial length adjustments for highly myopic eyes. Am. J. Ophthalmol. 223, 100–107. 10.1016/j.ajo.2020.09.019 32950507

[B3] Ejiyi C. J. Qin Z. Ukwuoma C. C. Nneji G. U. Monday H. N. Ejiyi M. B. (2024). Comparative performance analysis of boruta, SHAP, and borutashap for disease diagnosis: a study with multiple machine learning algorithms. Network 36 (3), 507–544. 10.1080/0954898X.2024.2331506 38511557

[B4] Gong D. Fang L. Cai Y. Chong I. Guo J. Yan Z. (2023). Development and evaluation of a risk prediction model for diabetes mellitus type 2 patients with vision-threatening diabetic retinopathy. Front. Endocrinol. (Lausanne) 14, 1244601. 10.3389/fendo.2023.1244601 37693352 PMC10484608

[B5] Guo D. He W. Wei L. Song Y. Qi J. Yao Y. (2023). The Zhu-Lu formula: a machine learning-based intraocular lens power calculation formula for highly myopic eyes. Eye Vis. (Lond). 10 (1), 26. 10.1186/s40662-023-00342-5 37259154 PMC10233923

[B6] Haigis W. Lege B. Miller N. Schneider B. (2000). Comparison of immersion ultrasound biometry and partial coherence interferometry for intraocular lens calculation according to haigis. Graefes Arch. Clin. Exp. Ophthalmol. 238 (9), 765–773. 10.1007/s004170000188 11045345

[B7] Hoffer K. J. Savini G. (2020). Update on intraocular lens power calculation study protocols: the better way to design and report clinical trials. Ophthalmology 128 (11), e115–e120. 10.1016/j.ophtha.2020.07.005 32653457

[B8] Holden B. A. Fricke T. R. Wilson D. A. Jong M. Naidoo K. S. Sankaridurg P. (2016). Global prevalence of myopia and high myopia and temporal trends from 2000 through 2050. Ophthalmology 123, 1036–1042. 10.1016/j.ophtha.2016.01.006 26875007

[B9] Jeong J. Song H. Lee J. K. Chuck R. S. Kwon J. W. (2017). The effect of ocular biometric factors on the accuracy of various IOL power calculation formulas. BMC Ophthalmol. 17 (1), 62. 10.1186/s12886-017-0454-y 28464806 PMC5414130

[B10] Jonas J. B. Jonas R. A. Bikbov M. M. Wang Y. X. Panda-Jonas S. (2022). Myopia: histology, clinical features, and potential implications for the etiology of axial elongation. Prog. Retin Eye Res. 96, 101156. 10.1016/j.preteyeres.2022.101156 36585290

[B11] Lei Q. Tu H. Feng X. Ortega-Usobiaga J. Cao D. Wang Y. (2021). Distribution of ocular biometric parameters and optimal model of anterior chamber depth regression in 28,709 adult cataract patients in China using swept-source optical biometry. BMC Ophthalmol. 21 (1), 178. 10.1186/s12886-021-01932-4 33849464 PMC8045194

[B12] Li T. Yang K. Stein J. D. Nallasamy N. (2020). Gradient boosting decision tree algorithm for the prediction of postoperative intraocular lens position in cataract surgery. Transl. Vis. Sci. Technol. 9 (13), 38. 10.1167/tvst.9.13.38 33384892 PMC7757635

[B13] Li L. Xiao K. Ma H. Lin J. Lin S. Lin X. (2025). Application and evaluation of virtual simulation technology in “corneal contact lens” education. BMC Med. Educ. 25 (1), 357. 10.1186/s12909-024-06378-y 40059152 PMC11892242

[B14] Lou W. Zhou W. Wu M. Jin H. (2024). A new intraocular lens power formula integrating an artificial intelligence-powered estimation for effective lens position based on Chinese eyes. Transl. Vis. Sci. Technol. 13 (10), 40. 10.1167/tvst.13.10.40 39476087 PMC11534016

[B15] Lu W. Xiao K. Zhang X. Wang Y. Chen W. Wang X. (2025). A machine learning model for predicting anatomical response to Anti-VEGF therapy in diabetic macular edema. Front. Cell Dev. Biol. 13, 1603958. 10.3389/fcell.2025.1603958 40519258 PMC12162914

[B16] Mita N. Yamazaki M. Seki Y. Sasaki Y. Shibuya E. Mito T. (2024). Prediction of low-addition segmented refractive intraocular lens position and deviation using anterior-segment optical coherence tomography. PLoS One 19 (6), e0305076. 10.1371/journal.pone.0305076 38857255 PMC11164357

[B17] Mo E. Feng K. Li Q. Xu J. Cen J. Li J. (2023). Efficacy of corneal curvature on the accuracy of 8 intraocular lens power calculation formulas in 302 highly myopic eyes. J. Cataract. Refract Surg. 49 (12), 1195–1200. 10.1097/j.jcrs.0000000000001303 37702529

[B18] Mo E. R. Chen Z. Feng K. E. Zhu Z. Xu J. Zhu C. (2024). Accuracy of modern intraocular lens formulas in highly myopic eyes implanted with plate-haptic intraocular lenses. Am. J. Ophthalmol. 265, 105–116. 10.1016/j.ajo.2024.04.017 38703800

[B19] Ning X. Yang Y. Yan H. Zhang J. (2019). Anterior chamber depth - a predictor of refractive outcomes after age-related cataract surgery. BMC Ophthalmol. 19 (1), 134. 10.1186/s12886-019-1144-8 31238910 PMC6591866

[B20] Norrby S. (2008). Sources of error in intraocular lens power calculation. J. Cataract. Refract Surg. 34 (3), 368–376. 10.1016/j.jcrs.2007.10.031 18299059

[B21] Olsen T. (2006). Prediction of the effective postoperative (intraocular lens) anterior chamber depth. J. Cataract. Refract Surg. 32 (3), 419–424. 10.1016/j.jcrs.2005.12.139 16631049

[B22] Pan C. W. Cheng C. Y. Saw S. M. Wang J. J. Wong T. Y. (2013). Myopia and age-related cataract: a systematic review and meta-analysis. Am. J. Ophthalmol. 156 (5), 1021–1033.e1. 10.1016/j.ajo.2013.06.005 23938120

[B23] Ponce-Bobadilla A. V. Schmitt V. Maier C. S. Mensing S. Stodtmann S. (2024). Practical guide to SHAP analysis: explaining supervised machine learning model predictions in drug development. CTS Clin. Transl. Sci. 17, e70056. 10.1111/cts.70056 39463176 PMC11513550

[B24] Qi J. He W. Zhang K. Guo D. Du Y. Lu Y. (2023). Actual lens positions of three intraocular lenses in highly myopic eyes: an ultrasound biomicroscopy-based study. Br. J. Ophthalmol. 108 (1), 45–50. 10.1136/bjo-2022-322037 36351786

[B25] Retzlaff J. A. Sanders D. R. Kraff M. C. (1990). Development of the SRK/T intraocular lens implant power calculation formula. J. Cataract. Refract Surg. 16 (3), 333–340. 10.1016/s0886-3350(13)80705-5 2355321

[B26] Saputro D. Wahyu N. Widyaningsih Y. (2025). Performance of ridge regression, least absolute shrinkage and selection operator, and elastic net in overcoming multicollinearity. J. Multidiscip. Appl. Nat. Sci. 5, 370–382. 10.47352/jmans.2774-3047.251

[B27] Shan M. Dong Y. Chen J. Su Q. Wan Y. (2022). Global tendency and frontiers of research on myopia from 1900 to 2020: a bibliometrics analysis. Front. Public Health 10, 846601. 10.3389/fpubh.2022.846601 35359777 PMC8960427

[B28] Sheard R. M. Smith G. T. Cooke D. L. (2010). Improving the prediction accuracy of the SRK/T formula: the T2 formula. J. Cataract. Refract Surg. 36 (11), 1829–1834. 10.1016/j.jcrs.2010.05.031 21029888

[B29] Shen X. Chen Z. Jia W. Wang Y. Chen T. Sun Y. (2024). Influencing factors of effective lens position in patients with Marfan syndrome and ectopia lentis. Br. J. Ophthalmol. 108 (12), 1634–1641. 10.1136/bjo-2023-325017 38604620

[B30] Taroni L. Hoffer K. J. Pellegrini M. Lupardi E. Savini G. (2023). Comparison of the new hoffer QST with 4 modern accurate formulas. J. Cataract. Refract Surg. 49 (4), 378–384. 10.1097/j.jcrs.0000000000001126 36729423

[B31] Tibshirani R. (2011). Regression shrinkage and selection via the lasso: a retrospective. J. R. Stat. Soc. Ser. B Stat. Methodol. 73 (3), 273–282. 10.1111/j.1467-9868.2011.00771.x

[B32] Ting D. S. W. Pasquale L. R. Peng L. Campbell J. P. Lee A. Y. Raman R. (2018). Artificial intelligence and deep learning in ophthalmology. Br. J. Ophthalmol. 103 (2), 167–175. 10.1136/bjophthalmol-2018-313173 30361278 PMC6362807

[B33] Ting D. S. J. Foo V. H. Yang L. W. Y. Sia J. T. Ang M. Lin H. (2021). Artificial intelligence for anterior segment diseases: emerging applications in ophthalmology. Br. J. Ophthalmol. 105 (2), 158–168. 10.1136/bjophthalmol-2019-315651 32532762

[B34] Vanathi M. (2024). Lens sizing calculation in phakic lens implantation - what is the best applicable measurement? Indian J. Ophthalmol. 72, 923–924. 10.4103/IJO.IJO_1402_24 38905456 PMC11329818

[B35] Vilaltella M. Cid-Bertomeu P. Serés-Noriega T. Huerva V. (2025). Accuracy of 12 IOL power calculation formulas in highly myopic eyes. Int. Ophthalmol. 45 (1), 264. 10.1007/s10792-025-03608-0 40560233 PMC12198256

[B36] Wei L. He W. Meng J. Qian D. Lu Y. Zhu X. (2021). Evaluation of the white-to-white distance in 39,986 Chinese cataractous eyes. Invest. Ophthalmol. Vis. Sci. 62 (1), 7. 10.1167/iovs.62.1.7 33393973 PMC7794278

[B37] Werner L. Izak A. M. Pandey S. K. Apple D. J. Trivedi R. H. Schmidbauer J. M. (2004). Correlation between different measurements within the eye relative to phakic intraocular lens implantation. J. Cataract. Refract Surg. 30, 1982–1988. 10.1016/j.jcrs.2003.10.041 15342066

[B38] Xu G. Wu G. Du Z. Zhu S. Guo Y. Yu H. (2021). Distribution of white-to-white corneal diameter and anterior chamber depth in Chinese myopic patients. Front. Med. (Lausanne) 8, 732719. 10.3389/fmed.2021.732719 34869427 PMC8639187

[B39] Yi C. Niu G. Zhang Y. Rao J. Liu G. Yang W. (2024). Advances in artificial intelligence in thyroid-associated ophthalmopathy. Front. Endocrinol. (Lausanne) 15, 1356055. 10.3389/fendo.2024.1356055 38715793 PMC11075148

[B40] Yoo Y. S. Whang W. J. Kim H. S. Joo C. K. Yoon G. (2019). Preoperative biometric measurements with anterior segment optical coherence tomography and prediction of postoperative intraocular lens position. Medicine 98 (50), e18026. 10.1097/MD.0000000000018026 31852065 PMC6922509

[B41] Yuan L. Kang D. Dong X. Liu L. Grzybowski A. Jin K. (2025). Artificial intelligence in clinical education in ophthalmology: a systematic review. VNS 42 (1), 0. 10.48130/vns-0025-0025

[B42] Zhao J. Lu Y. Zhu S. Li K. Jiang Q. Yang W. (2022). Systematic bibliometric and visualized analysis of research hotspots and trends on the application of artificial intelligence in ophthalmic disease diagnosis. Front. Pharmacol. 13, 930520. 10.3389/fphar.2022.930520 35754490 PMC9214201

[B43] Zhu S. Zhan H. Yan Z. Wu M. Zheng B. Xu S. (2023). Prediction of spherical equivalent refraction and axial length in children based on machine learning. Indian J. Ophthalmol. 71 (5), 2115–2131. 10.4103/IJO.IJO_2989_22 37203092 PMC10391375

