# Supplementary material for: An explainable machine learning framework for accurate prediction of postoperative anterior chamber depth in highly myopic cataract surgery
Source: Front Cell Dev Biol. 2026 Jul 7;14:1876904. doi: 10.3389/fcell.2026.1876904 (PMC13385035; doi:10.3389/fcell.2026.1876904)
Supplement: Supplementary file 1 [file Image1.pdf]

Supplementary Figure 1. Performance of the Random Forest model in the one-eye-per-patient sensitivity analysis.

A. Prediction accuracy and correlation analysis between the actual and predicted Postop_ACD.

B. Bland–Altman plot showing the agreement between the actual and predicted Postop_ACD values. The solid line represents the mean difference, and the dashed lines indicate the 95% limits of agreement.

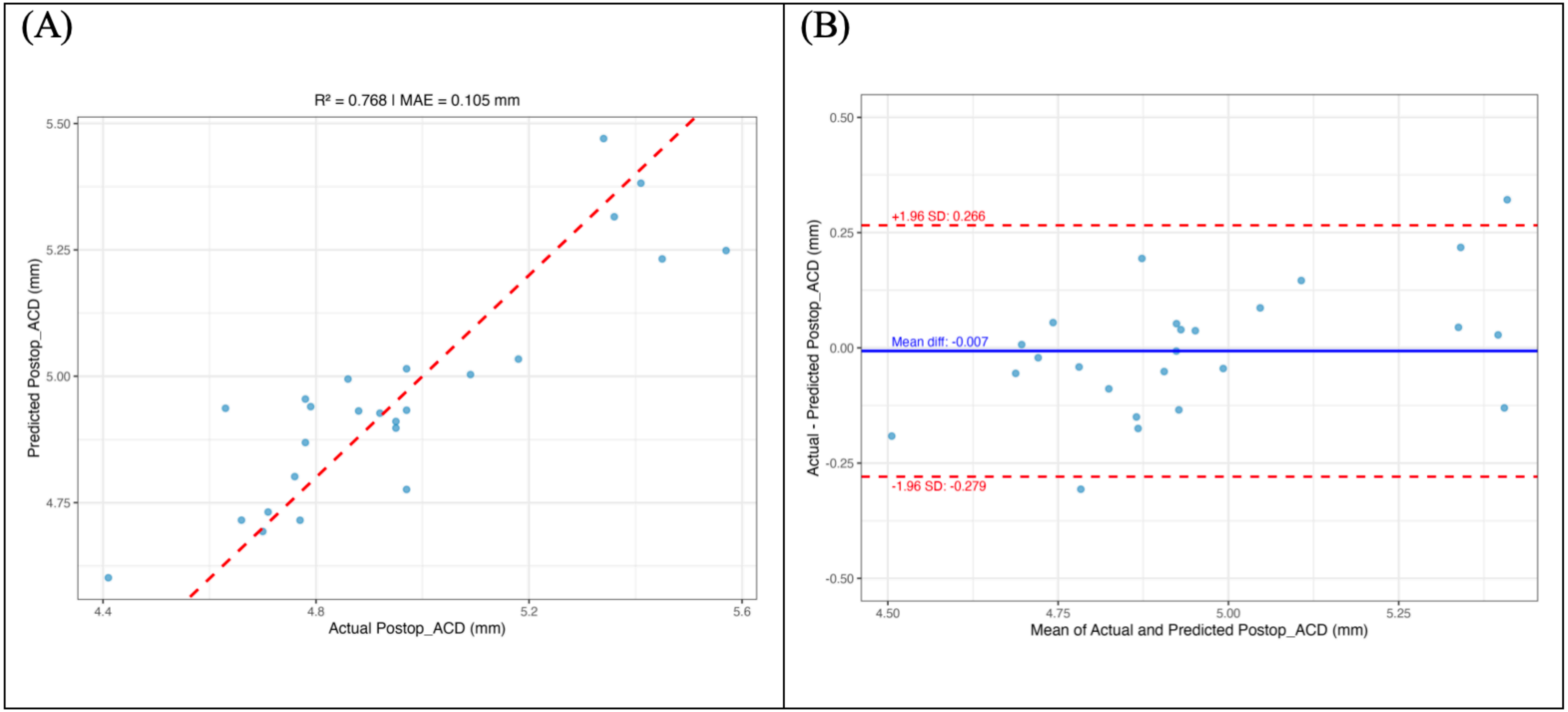

Abbreviations:

ACD = anterior chamber depth; R^2 = coefficient of determination; MAE = mean absolute error
